# Development of a two-current choice flume behavioural bioassay for juvenile *Panulirus ornatus* response to moulting cues

**DOI:** 10.1038/s41598-022-25969-7

**Published:** 2022-12-12

**Authors:** Tara R. Kelly, Quinn P. Fitzgibbon, Dean R. Giosio, Andrew J. Trotter, Gregory G. Smith

**Affiliations:** 1 Institute for Marine and Antarctic Studies (IMAS), University of Tasmania, Private Bag 49, Hobart, TAS 7001 Australia; 2 School of Engineering, University of Tasmania, Hobart, TAS 7000 Australia

**Keywords:** Behavioural methods, Animal behaviour

## Abstract

Characterising crustacean behaviour in response to conspecific chemical cues contributes to our evolving knowledge of the drivers of their social behaviour. There is particular interest in understanding the chemical and behavioural mechanisms contributing to cannibalism at ecdysis, as this behaviour substantially limits culture productivity of several commercially important crustaceans. Before investigating the role of chemoreception in cannibalism of moulting crustaceans, we must investigate its role in detecting moulting conspecifics. Here we use a two-current choice flume to observe juvenile tropical rock lobster (*Panulirus ornatus*) behavioural response to conspecific moulting cues and identifying attracted and avoidant behaviours correlating to moult stage and social relationship. Observed cue preferences show inter-moult juveniles are attracted to the moulting cues of lobsters to which they are socially naïve. In contrast, post-moult and inter-moult juveniles avoid the moulting cues of individuals whom they are socially familiar with. Average speed and total distance travelled by lobsters increases in response to conspecific moulting cues. This study demonstrates the suitability of a two-current choice flume for behavioural assays in *P. ornatus* and characterises clear behavioural patterns in juveniles exposed to conspecific moulting cues. This provides important framework for understanding the role of chemical communication in eliciting cannibalism.

## Introduction

Olfaction is well evolved in crustaceans to detect changes within their chemosensory rich environment^1^. The coevolution of signal emission and reception in crustaceans has shaped courtship behaviours^2–4^, behavioural responses to injured or diseased conspecifics^5–8^, clustering and sheltering behaviour^9,10^, and social hierarchies^11,12^. Whilst chemoreceptors cover a large majority of the spiny lobster body, olfaction is facilitated by aesthetasc chemosensilla, located on the distal end of the lateral flagellum of the antennules, which are innervated with olfactory receptor neurons^13,14^. The aesthetascs feature setae which are permeable to exogenous chemicals and play a role in gauging the concentration and direction of odorants^15^. Olfactory signals play a large role in communication between crustaceans, including communication of dominance and moult stage in crayfish^11,16–19^. Whilst physical contact, particularly antennal touching, appears to be ubiquitous in low intensity aggressive encounters in the spiny lobster, *Jasus edwardsii*, spontaneous, high intensity attacks are not preceded by such contact^20^. The lack of a physical precursor indicates chemical communication is contributing to some aggressive encounters.

Odours from injured conspecifics play a role in triggering opportunistic cannibalism and exuvia consumption. Exuvia consumption is seen in several crustacean species and is commonly reported under various spiny lobster communal rearing conditions, with some suggesting this behaviour improves growth as an additional nutrient source^21–23^. Hermit crabs, *Clibanarius digueti* and *Paguristes perrieri,* known to be cannibalistic and carrion scavengers, are attracted to conspecific and heterospecific injury cues and increase foraging behaviour in response to this cue^24^. Conversely, the odour of injured or diseased conspecifics reduces foraging behaviour in the spiny lobster, *Panulirus argus,* and the blue crab, *Callinectes sapidus*, who avoid injured individuals^6–8,25,26^. These are highly cannibalistic species displaying a risk-averse behaviour, rather than opportunistic cannibalism. The prevalence of cannibalism is recognised as one of the most significant limiting factors in the culture productivity of many commercially important crustaceans, including spiny lobsters^27–31^. The moult cycle is essential for the growth and development of most crustaceans, however, cannibalism in marine and freshwater crustaceans typically occurs at ecdysis, when animals are physically most vulnerable due to the loss of their protective exoskeleton. Previous research has indicated that water borne chemical cues released during ecdysis may be recognised by conspecifics, therefore playing a role in mediating cannibalism^18,30,32–34^.

Here we investigate the behavioural response of tropical rock lobster juveniles, *Panulirus ornatus,* to chemical cues released by moulting conspecific lobsters. *Panulirus ornatus* is an emerging candidate for commercial aquaculture given its high international demand, value and fast growth^27,35^. Whilst their social behaviours in the wild are not well documented, *P. ornatus* juveniles are a highly gregarious species in culture, displaying a strong cannibalistic nature during early juvenile development when ecdysis occurs most frequently. Not only do they opportunistically cannibalise deceased conspecifics (secondary cannibalism), they will attack and cannibalise live, moulting individuals (primary cannibalism), which presents a significant constraint to the viability of commercial production^22^. Establishing a method to identify odour mediated behavioural patterns and classifying these behaviours is essential in establishing the role of chemical communication in cannibalism.

Methods, such as counter-current troughs, Y-mazes, and shuttle box systems have been used to assess fish behaviour in relation to conspecific recognition, environmental pressures, and preference for chemical cues, such as toxins and chemoattractants^36–43^. These systems are potentially simple to build and can produce natural cue gradients, however they offer poor control of cue plumes and the movement of response animals is often restricted^44^. Two-current choice flumes use a series of honeycomb collimators to separate and evenly distribute cues within a single open space, or choice arena, allowing an animal free movement between cues. Thus, two-current choice flumes are ideal for the study of chemosensory behaviour, such as preference to a chemical cue. Laminar flow, reduced animal stress, experiment duration, eliminating bias, and appropriate utilisation and analysis of data, are key areas of focus to produce reliable and replicable results when using choice flumes^44^.

Research in the crayfish *Procambarus clarkii,* has seen naïve individuals change behaviour when exposed to odours from dominant and submissive conspecifics in a Y-maze choice-flow-channel, and vice versa, indicating recognition of individuals and their dominance status without physical or visual contact^17^. Additionally, the rusty crayfish, *Orconectes rusticus,* is able to distinguish, through chemical cues alone, between moulting individuals and food cues, and show an attraction to recently moulted crayfish^18^. In this study we examine the behavioural response of inter-moult and post-moult lobsters to moulting cues from socially naïve and familiar conspecifics using a two-current choice flume. Our study aims to apply the standards set out by Jutfelt, Sundin^44^ to develop a replicable assay using a two-current choice flume and identify patterns of juvenile preference to conditioned water containing conspecific moulting cues. We hypothesise this assay will reveal a correlation between moult stage, social relationship, and attraction to conspecific moulting cues, ultimately identifying factors to be addressed in future research focused on the mechanisms responsible for triggering cannibalism during the moult. This will provide a model for the examination of mitigating factors which may limit cannibalism in commercial culture.

## Materials and methods

### Experimental lobsters

*Panulirus ornatus* individuals were reared from eggs at the Institute for Marine and Antarctic Studies (IMAS) following procedures of Ikeda, Smith^45^, Fitzgibbon and Battaglene^46^ and Fitzgibbon and Battaglene^47^. Juveniles (instar 4) were housed in groups of 6–10 individuals within five 18 L opaque plastic tanks (n = 40). Polyvinyl chloride (PVC) tubes lined with oyster mesh were provided for shelter. Water quality was monitored daily and maintained at an average temperature of 27.8 °C ± 0.06, dissolved oxygen 101.8% ± 0.2, salinity 34.1 ppt ± 0.06, and pH 8.10 ± 0.01. Tanks were syphoned twice daily to remove lobster waste and uneaten feed. Lobsters were fed IMAS commercial-in-confidence formulated feed, at 5% of total tank biomass split into six feeds over 24 h. Housing tanks were kept under a 14:10 L:D photoperiod, with blue LED light providing the day phase. Each lobster was ID tagged by adhering a numbered piece of waterproof paper to their dorsal carapace using gel super glue. Individual tagging of lobsters allowed for moult staging, which classified juveniles as inter-moult, post-moult, or pre-moult. Intermoult lobsters were within 30–67% (mean 46.3% ± 4) way through their current moult cycle. Post-moult lobsters had moulted within 24–36 h prior to their use in an experiment replicate, making them approximately 4.5–15% (mean 8.37% ± 1.3) of the way into their current moult cycle. Average moult interval was 13.2 ± 0.4 days. Pre-moult juveniles were identified by the presence of a dark suture line visible on the dorsal and anterior edges of the gill cover up to 18 h before ecdysis (Supplementary Fig. 1). Individual’s sex was identified by the genital pores on the third leg of females, and fifth leg of males. Lobsters total wet weight (g) and moult stage were recorded throughout the experiment. Housing lobsters in isolated groups allowed for experimental lobsters to be familiar to one another, or naïve to one another.

### Choice flume

**Figure 1:**
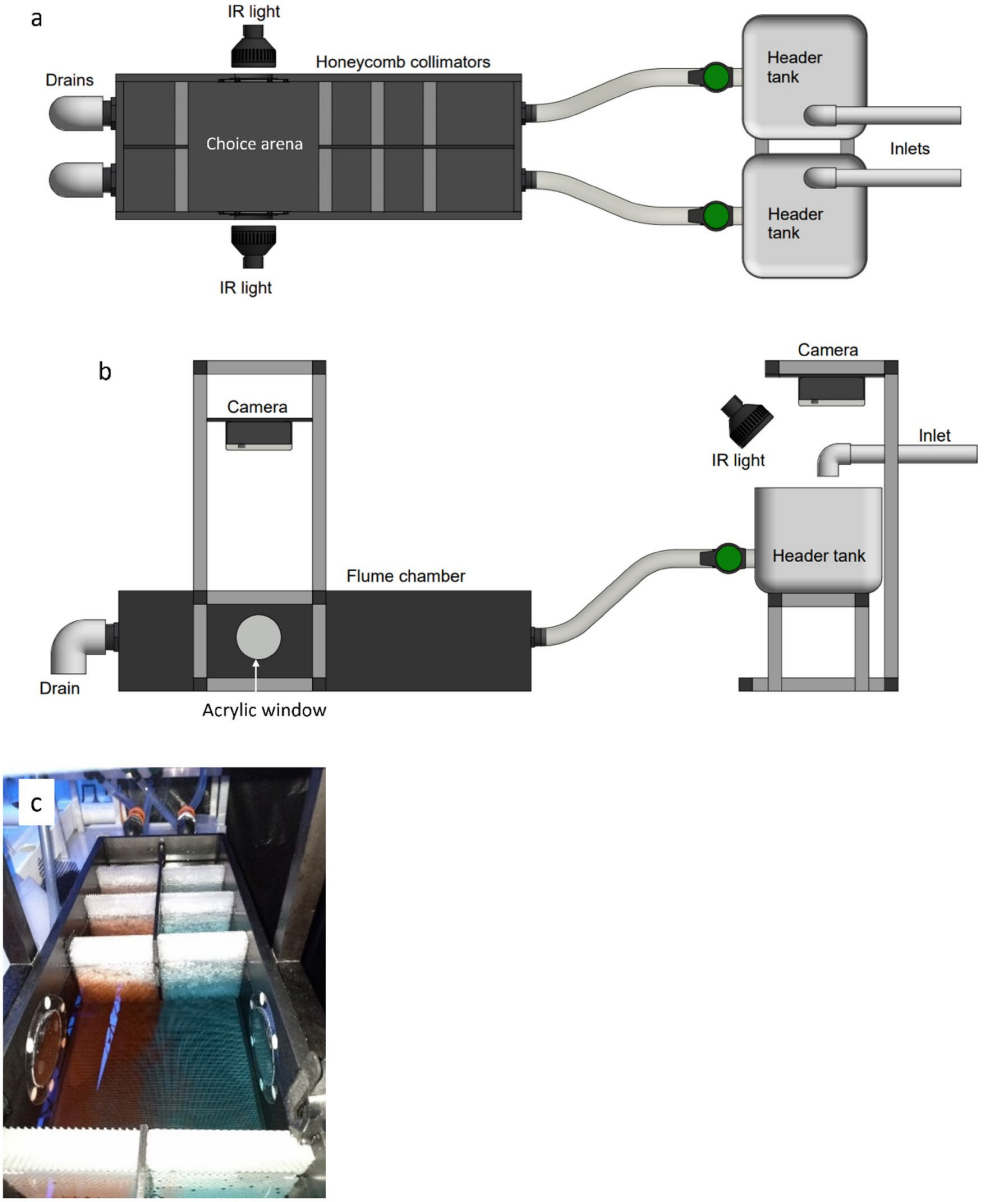
(**a**) Top-down schematic of two-current choice flume used for this study. Water enters both header tanks at 2 L min^−1^, then flows into main flume chamber, passing through three layers of honeycomb collimators to produce two distinct laminar currents in the choice arena. The choice arena is illuminated by two infrared lights placed at clear acrylic windows. (**b**) Side view schematic. Cameras in waterproof housing are secured to support stands and located directly above the header tanks and the choice arena. The header tanks are illuminated by a single infrared light placed at an angle to minimise surface reflection. (**c**) Demonstration of laminar flow in choice arena using red and blue dye, flow rate of 2 L min^−1^.

The design and testing of a choice flume was completed based on recommendations by Jutfelt, Sundin^44^. The flume was constructed with black high-density polyethylene featuring a 250 × 250 mm choice arena, designed to comfortably hold a single juvenile up to the sixth instar (Fig. 1a). Infrared LED light sources (6 × 3 W) placed at two clear acrylic ports flush with the internal wall surface of the choice arena provided illumination without surface reflection (Fig. 1a,b). Infrared light does not interrupt regular nocturnal feeding and foraging behaviours of *P. ornatus*^47,48^*.* Two custom-built video camera units (5 MP infrared sensitive camera module with adjustable focus lens) with internal SSD storage and water-proof housing (IP68) were assembled above the flume system to allow recordings of both the choice arena and header tanks.

Water was supplied to two header tanks (2 L min^−1^ each) positioned upstream of the main flume tank (Fig. 1b). Water flow from the header tanks to the main flume tank was adjustable via taps. Laminar flow in the choice arena was achieved through equal flow rate in both currents and the addition of three layers of honeycomb collimators arranged from upstream to downstream (4 mm diameter, 20 mm thickness), (3.5 mm diameter, 20 mm thickness), (3.5 mm diameter, 25 mm thickness). The angle of elbow pipes fitted to outflow openings controlled the water level in the choice arena and ensured water was drawn evenly from both currents. Laminar flow was tested by adding coloured food dye to the supply header tanks and showed limited mixing of water flows within the choice arena (Fig. 1c). A dye test validated laminar flow before all experimental runs. All tank elements were thoroughly cleaned between replicates. Removable pieces, such as honeycomb collimators and plastic tubing, were submerged in a chlorine bath (sodium hypochlorite 50 ppm) for 4 h, then rinsed in saltwater. The flume and header tanks were drained, rinsed with diluted chlorine, and flushed with saltwater. Water quality in the choice flume system was monitored before and after each experiment and maintained at an average temperature of 26.0 °C ± 0.3, dissolved oxygen 102.02% ± 0.6, salinity 34.08 ppt ± 0.1, and pH 8.07 ± 0.02.

### Experimental protocol

Experimental controls examined an inter-moult juvenile lobster in the choice arena and no lobsters in either header tank (n = 4, Table 1). Negative controls examined an inter-moult juvenile in the choice arena and an inter-moult juvenile in one randomly selected header tank (n = 3). This negative control is designed to detect confounding and false conclusions about the type of conspecific cue (inter-moult cues or moulting cues) mediating observed behaviour*.* Treatment replicates consisted of an inter-moult, or post-moult juvenile placed in the choice arena, and a pre-moult juvenile in one randomly selected header tank (n = 5–6). Lobsters were transferred from housing tanks to the flume system in jugs of saltwater (2 L), reducing handling and air exposure stress. Lobsters were transferred to the choice arena and header tank at 1600 h, approximately 40 min before the dark phase began, and 1 h before recording (1 fps) began, to acclimatise lobsters to the experimental environment. Experiments were conducted during the dark phase as *P. ornatus* moult and feed most commonly at night.

**Table 1:** *P. ornatus,* tropical rock lobster, moult stages and social relationship in control and treatment replicates.

Experiment replicates				
Group	Header tank lobster moult stage	Choice arena lobster moult stage	Social relationship	n
Inter-moult/familiar	Pre-moult	Inter-moult	Familiar	5
Inter-moult/naïve	Pre-moult	Inter-moult	Naïve	6
Post-moult/familiar	Pre-moult	1-day post-moult	Familiar	5
Post-moult/naïve	Pre-moult	1-day post-moult	Naïve	6
Experimental control	None	Inter-moult	N/A	4
Negative control	Inter-moult	Inter-moult	Familiar	3

n = number of replicates.

### Tracking

Images (JPG) were written to AVI format on MATLAB R2020b, with a frame rate of 6 fps. The ImageJ plugin, AnimalTracker, was used to track the activity of lobsters within the two laminar currents, which were designated as regions of interest using AnimalTracker Zone Designer^49,50^. Each frame was filtered using AnimalTracker’s inbuilt background subtraction and thresholding, then postprocessed to remove excess noise (Supplementary Fig. 2). A single reference point on lobsters was followed by AnimalTracker, without concern for body orientation, to produce longitude and latitude coordinates for each frame (Supplementary Fig. 3 and Supplementary Video 1). Tracks were manually edited if tracker lost the reference point on the body for several frames. For each control and experimental replicate three hours of observation was tracked using this method. A baseline observation hour was analysed for all replicates from 1.5 h after the commencement of the dark phase. Lobsters in the choice arena during treatment replicates were tracked for 1 h before and 1 h after the lobster in the header tank moulted. Lobsters in the choice arena during control replicates were analysed for 1 h either side of the mean moult time observed in treatment replicates (5 h after commencement of dark phase).

AnimalTracker Tracking Analyzer was used to analyse the coordinate points of lobsters in the choice arena and calculate their time spent in each laminar current. The average speed and travel distance of individual lobsters was calculated by comparing coordinate points between consecutive frames. When the camera unit overheated and exceeded 80 °C due to long hours of use within a sealed container it would pause capture to cool, resulting in large gaps in the header tank recordings for five of 22 treatment replicates. This prevented accurate speed and distance measurements; hence these five replicates were excluded when analysing average speed and travel distance of lobsters in the header tank.

### Statistical analyses

Data were assessed for normality and homoscedasticity with the Shapiro–Wilk’s and Levene’s tests.

A t-test was used to compare mean values of speed, distance, and moult time. Three analysis periods were chosen: a baseline hour covering 1.5–2.5 h after beginning of dark phase, 1 h before upstream ecdysis occurs, and 1 h following upstream moult. Tracking data were analysed in 10 min blocks. For each 10 min block the total time spent in both choice arena currents was calculated as a percentage. Coordinate data were compared across consecutive image frames to produce a value for total distance covered (cm) by lobsters, as well as their average speed (cm/s) for each 10 min block. One-way ANOVA was used to compare mean time spent in a particular current during 10 min exposure to moult cues across the four treatment groups, followed by multiple t-tests with Bonferroni correction (α = 0.008). Welch’s t-test for unequal variances was used to compare mean values of time spent in currents between control groups, and lobster wet weight between all experimental groups. The Mann–Whitney U test was used to assess measures of speed for nonnormal data with unequal variance. One-way ANOVA was used to compare means of average speed and distance of choice arena lobsters, among the four treatment groups.

The non-parametric Friedman test for dependent samples was used to compare average speed and total distance covered by lobsters across three analysis hours, followed by pairwise comparisons using Wilcoxon signed-rank test with a Bonferroni correction (α = 0.0167). Chi square test was used to test for significance between preference patterns and sex, and preference patterns and control types. Mean values are presented with the standard error of the mean (mean ± sem) unless otherwise stated. Statistical analyses were conducted using R Studio (R Core^51^) and Microsoft Excel^52^.

## Results

### Validation of controls

Flume current preferences between experimental and negative control groups were not considerably different during the baseline observation hour (Welch’s t-test, t(4) = − 0.04, *P* = 1.0), and 1 h before the mean moult time (Welch’s t-test, t(4) = − 0.008, *P* = 1.0)(Table 2). Indicating, current preference is similar for water conditioned with an inter-moult conspecific and non-conditioned water.

**Table 2:** Mean percentage time lobsters spent in the conspecific conditioned current of the choice arena for four treatment groups and two control groups.

Mean percentage time	n	Baseline hour	1 h before moult	10 min moult cue exposure
Inter-moult/naïve	6	34.9 ± 10	44.7 ± 12	88.9 ± 5^b^
Inter-moult/familiar	5	45.9 ± 19	27.0 ± 14	21.9 ± 6^a^
Post-moult/naïve	6	46.8 ± 17	52.7 ± 15	47.3 ± 14^ab^
Post-moult/familiar	5	35.6 ± 13	32.5 ± 15	27.1 ± 12^a^
Experimental control	4	42.0 ± 17	*52.6 ± 16	
Negative control	3	42.9 ± 6	*53.0 ± 10	
Experimental total	29	41.2 ± 6	42.8 ± 6	

Mean values ± sem. n = number of replicates per treatment. *Analysis at 1 h before mean moult time. No upstream moults occurred in controls treatments. Lower case letters denote statistically similar treatment groups during 10 min moult cue exposure.

For control replicates, no difference was seen in average speed of lobsters in the choice arena across the three analysis hours (Friedman test, Q = 2.0, 2df, *P* = 0.37) and no difference was seen in the total distance travelled during the three analysis hours (Friedman test, Q = 4.6, 2df, *P* = 0.10).

### Flume current preference

**Figure 2:**
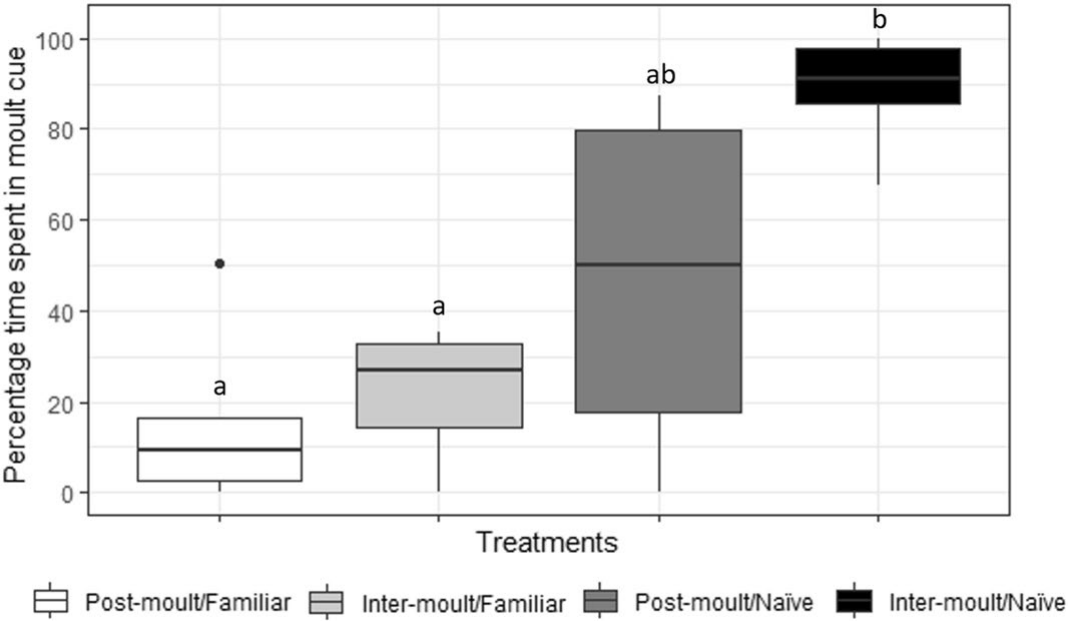
Mean percentage time juvenile *Panulirus ornatus* spend in choice arena current containing conspecific moulting cues during a 10 min exposure window. Four treatment groups denoted by the response lobsters moult-stage and relationship with moulting lobster, post-moult/familiar (n = 5), inter-moult/familiar (n = 5), post-moult/naïve (n = 6) and inter-moult/naïve (n = 6). Lower case letters indicate significant differences in percentage time spent in moult cues. Central black line in each box represents median value.

The baseline hour observed at the same time for all replicates shows no statistical difference in flume current preferences across treatment groups (One-way ANOVA, F_(4, 24)_ = 0.14, *P* = 0.97) with lobsters spending on average, 41.2% ± 6% of their time in one current of the choice arena (Table 2). Analysis of current preference during the 1 h before a lobster moults in the header tank (or the 1 h before the mean moult time for control replicates) also shows no difference between treatment groups (One-way ANOVA, F_(4, 24)_ = 0.58, *P* = 0.68) with an average 42.8% ± 6% of time spent in one current (Table 2). It takes approximately 12 min for water from two full header tanks to pass through the entire flume system. To assess the behavioural response of lobsters in the choice arena to conspecific moulting cues, their time in the moulting cue current was assessed for 10 min after a lobster began moulting in a header tank. A significant difference in flume current preference is seen during the 10 min following an upstream moult, during which time the choice arena lobsters are exposed to moult conditioned water (Fig. 2, One-way ANOVA, F_(3, 18)_ = 10.4, *P* = 0.0003). Inter-moult/naïve pairings spend a significantly greater amount of time in moult conditioned water compared to inter-moult/familiar and post-moult/familiar pairings (Table 2, Bonferroni corrected t-test, α = 0.008: inter-moult/naïve and inter-moult/familiar t(8) = 8.15, *P* = 0.00004, inter-moult/naïve and post-moult/familiar t(6) = 7.06, *P* = 0.0004).

### Attraction and avoidance response to moulting cues

**Figure 3:**
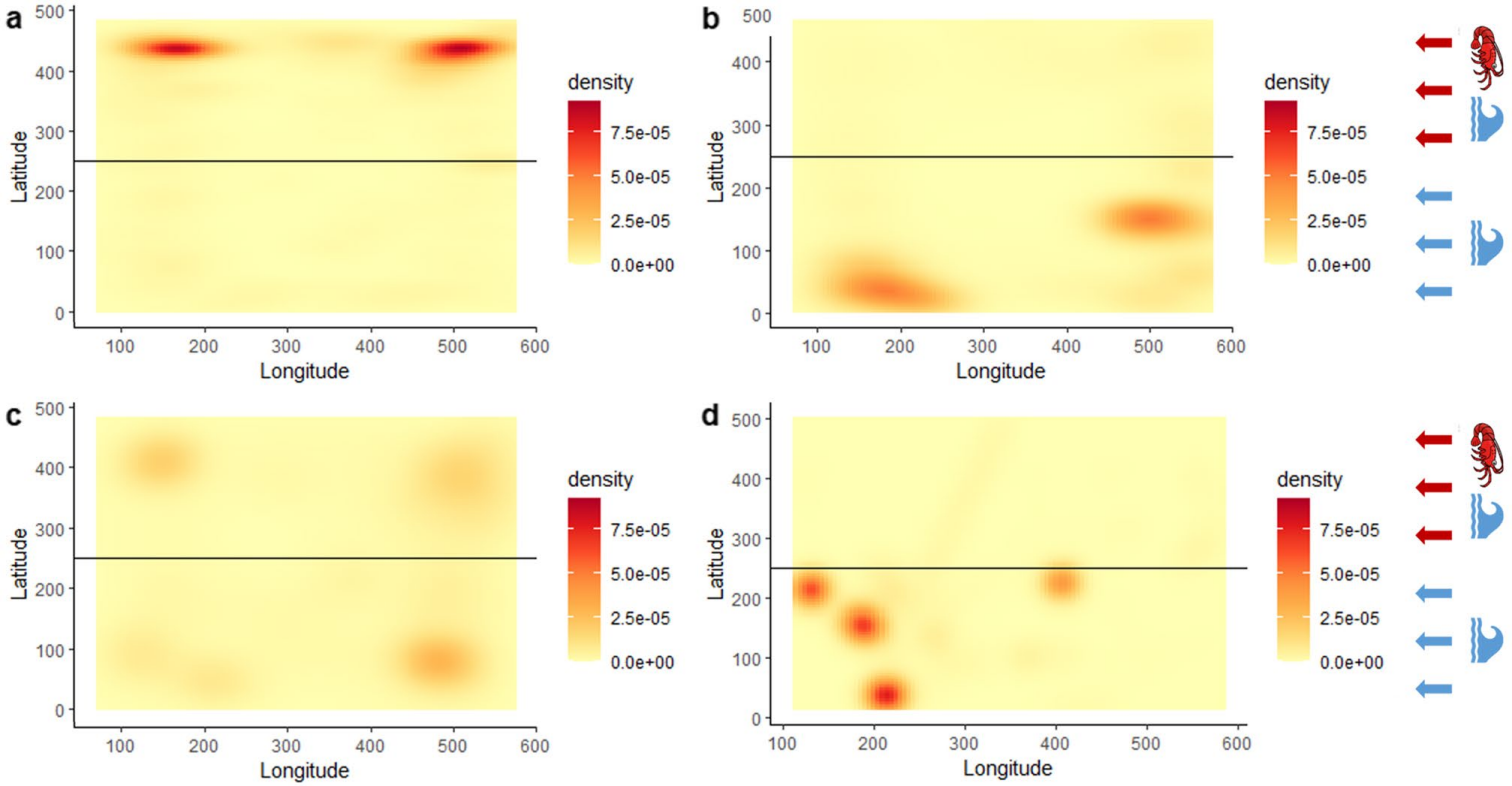
Density plot for number of detection points of lobster coordinate position in choice arena during 10 min exposure to conspecific moulting cues. One treatment group per density plot, (**a**) inter-moult/naïve (n = 6), (**b**) inter-moult/familiar (n = 5), (**c**) post-moult/naïve (n = 6), (**d**) post-moult/familiar (n = 5). In each plot conspecific cue current is present above the horizontal line and water only current is present below the line. Water flow is from right to left.

The greater average time spent in conspecific moulting cues by inter-moult/naïve lobsters (88.9% ± 5%, Table 2) indicates inter-moult lobsters are attracted to moulting cues released by lobsters they are naïve to. Conversely, both inter-moult and pre-moult lobsters were avoidant of moulting cues released by lobsters whom they are familiar with (21.9% ± 6% and 27.1% ± 12% respectively, Table 2). This behaviour is clear when mapping the coordinate positions of choice arena lobsters during exposure to conspecific moult cues (Fig. 3). Lobsters in the inter-moult/naïve treatment have the greatest coordinate density in the moulting cue current (above the horizontal line, Fig. 3a). Lobsters in the inter-moult/familiar and post-moult/familiar treatments avoid the moulting cue current and have the greatest coordinate density in the water only current (below the horizontal line, Fig. 3b,d). Coordinate density is spread across corners of the choice arena in post-moult/naïve replicates demonstrating a lack of preference for or against conspecific moulting cues for this treatment (Fig. 3c).

Whilst inter-moult/familiar and post-moult/familiar pairings did not have significantly different current preferences during the 1 h before upstream moult, both spent less time in the conspecific cue during this time compared to other treatments and went on to demonstrate significant avoidance to the moulting cue. The post-moult/naïve treatment group did not show a preference for or against the moulting cue current (47.3% ± 14%).

The sex of lobsters placed in the choice arena and header tank was either male–female (n = 6), male-male (n = 5), female-female (n = 6) or female-male (n = 5) respectively. The sex of these paired lobsters was not associated with attraction or avoidant preference for conspecific moulting cues (*X*^2^ = 2.0, 3df, *P* = 0.57). The average time, after commencement of the dark phase, that ecdysis occurred was 5.00 ± 0.4 h (min 2.07, max 8.85 h). The mean moult time of lobsters in the header tank is statistically similar between the four treatment groups (One-way ANOVA, F_(3, 18)_ = 1.0, *P* = 0.4) and between those which show attraction and avoidance to conspecific moulting cues (t-test, t(14) = 1.5, *P* = 0.15). No difference observed in mean wet weights (g) between header tank lobsters and choice arena lobsters (Welch’s t-test, t_(25)_ = − 1.9, *P* = 0.07) and the wet weight difference between the two experimental lobsters is statistically similar between treatment groups (One-way ANOVA, F_(3, 12)_ = 0.14, *P* = 0.94) and between attracted and avoidant treatment groups (t-test, t(9) = − 0.09, *P* = 0.93).

### Activity of choice arena lobsters

No statistically significant difference was found in the average speed of choice arena lobsters across treatment groups during the baseline hour (One-way ANOVA, F_(3, 18)_ = 0.16, *P* = 0.92), 1 h before upstream moult (One-way ANOVA, F_(3, 18)_ = 0.67, *P* = 0.58), and 1 h after upstream moult (One-way ANOVA, F_(3, 18)_ = 0.77, *P* = 0.52). Similarly, no significant difference in total distance covered by choice arena lobsters was seen between treatment groups during the baseline hour (One-way ANOVA, F_(3, 18)_ = 0.08, *P* = 0.97), 1 h before upstream moult (One-way ANOVA, F_(3, 18)_ = 1.7, *P* = 0.21), and 1 h after upstream moult (One-way ANOVA, F_(3, 18)_ = 0.93, *P* = 0.45).

**Figure 4:**
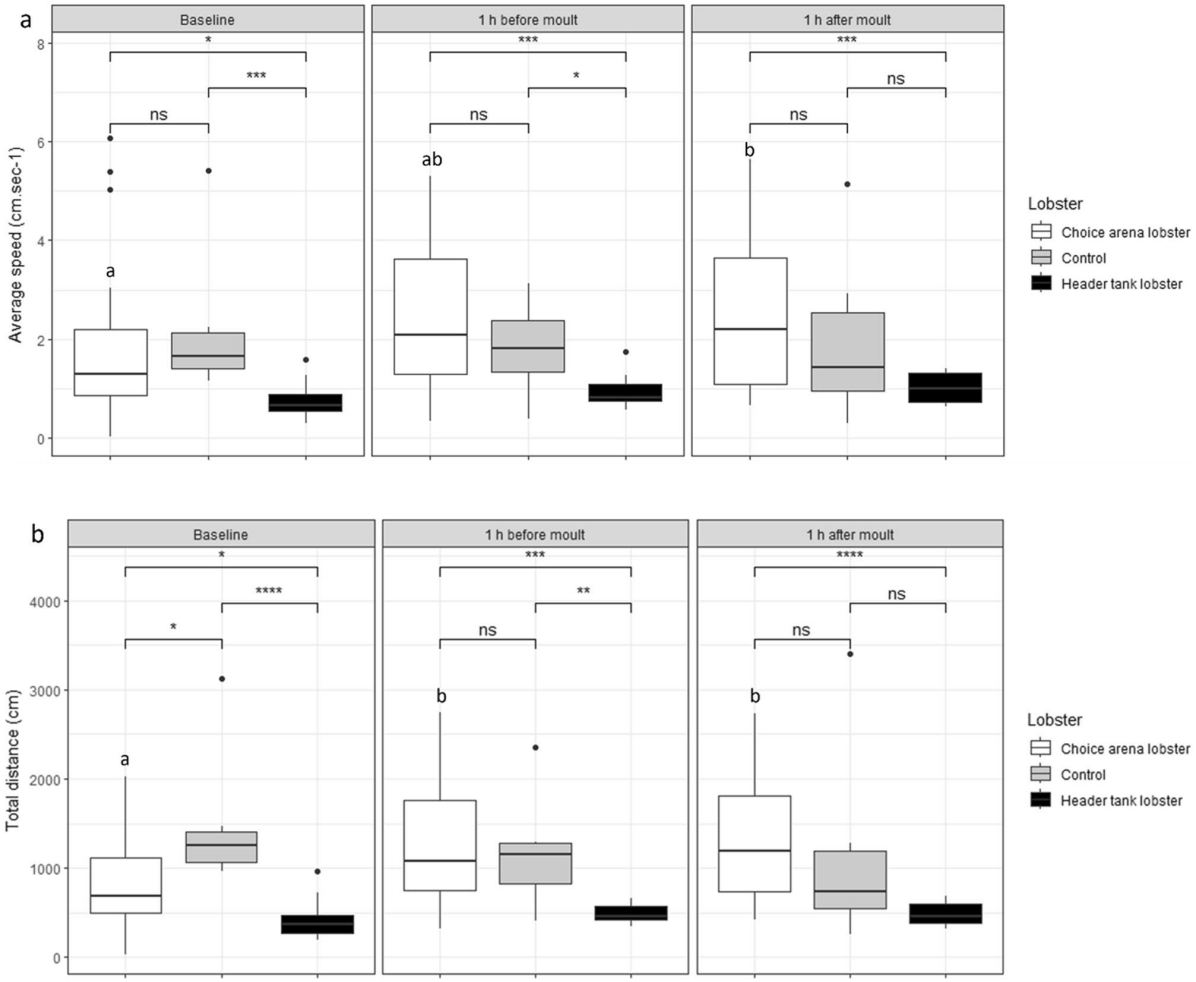
(**a**) Average speed of lobsters during control replciates (*Control*, n = 7), and during treatment replicates (*Header tank lobsters*, n = 17, and *Choice arena lobsters*, n = 22). (**b**) Total distance travelled by lobsters during control replciates (*Control*, n = 7), and during treatment replicates (*Header tank lobsters*, n = 17, and *Choice arena lobsters*, n = 22). Central black line in each box represents median value. Lower case letters indicate significant differences in choice arena lobsters across time-points (*P* < 0.01). Wilcoxon signed-rank test used to statistically compare groups. Bonferroni corrected alpha = 0.0167. ns *P* > 0.05, *P < 0.05, **P < 0.01, ***P < 0.001, ****P < 0.0001.

There is a significant difference in both the average speed and distance covered by lobsters in the choice arena over the three analysis hours (Friedman test, average speed Q = 8.27, 2df, *P* = 0.02, total distance Q = 13.36, 2df, *P* = 0.001). Post hoc analysis reveals the average speed of lobsters was significantly lower during the baseline hour compared to the 1 h after moult (Wilcoxon signed-rank test, α = 0.0167, Z = 33, *P* = 0.001, Fig. 4a). The significant difference in total distance travelled is due to differences seen between the baseline hour and 1 h before moult (Wilcoxon signed-rank test, α = 0.0167, Z = 46, *P* = 0.005), and between the baseline and 1 h after moult (Wilcoxon signed-rank test, α = 0.0167, Z = 18, *P* = 0.0002, Fig. 4b).

The average speed of choice arena lobsters in treatment replicates was not significantly different to the activity seen in control replicates across analysis hours (Mann–Whitney test, α = 0.0167, U = 597.5, *P* = 0.38, Fig. 4a). Additionally, there was no difference in total distance covered by choice arena lobsters across analysis hours when comparing controls and treatment groups (Mann–Whitney U test, α = 0.0167, U = 560, *P* = 0.24, Fig. 4b).

### Activity of moulting lobsters

No difference was seen in average speed of lobsters in the header tank across the three analysis hours (Friedman test, Q = 3.3, 2df, *P* = 0.19) and no difference was seen in the total distance travelled during the three analysis hours (Friedman test, Q = 2.2, 2df, *P* = 0.34).

Average speed of lobsters in the header tank was significantly less than lobsters in the choice arena during all analysis hours (Fig. 4a); baseline (Mann–Whitney U test, U = 76, *P* = 0.02), 1 h before moult (Mann–Whitney U test, U = 62, *P* = 0.0002), 1 h after moult (Mann–Whitney U test, U = 59, *P* = 0.0002). Distance travelled by lobsters in the header tank is significantly less than that of lobsters in the choice arena during all three analysis hours (Fig. 4b); baseline (Mann–Whitney U test, U = 62, *P* = 0.006), 1 h before moult (Mann–Whitney U test, U = 67, *P* = 0.0004), 1 h after moult (Mann–Whitney U test, U = 25, *P* = 0.00001).

## Discussion

To our knowledge, this is the first study to demonstrate distinct attraction and avoidance of moulting cues in the highly cannibalistic spiny lobster, *Panulirus ornatus*. A two-current choice flume bioassay produced clear behavioural observations and demonstrated that juvenile lobsters display distinct preferences for moulting cues depending on moult stage and relationship to moulting lobster. These findings provide insight into the biological and social factors influencing conspecific interaction during ecdysis, which is vital in understanding factors contributing to primary cannibalism of moulting juveniles. Identifying these factors is necessary to efforts mitigating cannibalism in *P. ornatus* culture production.

Previous investigations of moulting cues in lobsters has largely focused on identifying mating cues and behaviours in adults^53,54^. For many decapod crustaceans females moult prior to mating. Due to this cooccurrence, efforts have been made to identify chemical cues released by moulting adult females and find pheromones triggering male courtship behaviours in crabs^2,3,33^. These studies found males demonstrating courtship behaviours, such as shelter sharing, prior to female moult. Our examination of juvenile *P. ornatus* found behavioural preference for conspecific moulting cues is evident during exposure to a moult conditioned water current. It is understandable that the response behaviours observed in our study differ from those using adult crustaceans, as the juveniles used here are not producing sex-specific adult hormones. A lack of research into *P. ornatus* moult cues and the behaviours they elicit in juvenile conspecifics has directed our research in order to assess these behaviours during this particularly cannibalistic early juvenile life-stage.

Chemical cues aid crustaceans in various social behaviours, such as aggregation^9,10^, differentiating between familiar and naïve individuals^17,55^, as well as between dominant and subordinate individuals^11,12,17,56^ and between inter-moult and moulting lobsters^18,33^. Our findings align with crayfish research, showing they are capable of distinguishing conspecific moulting cues and display increased activity in response^18^. Both pre-moult and inter-moult lobsters have a distinct avoidant response to a familiar conspecific moulting cue. This indicates a recognition of familiar cues from a lobster with whom they are usually housed. The attracted response seen in inter-moult/naïve pairings may be indicative of searching behaviour in response to an unknown cue. This distinction between familiar and naïve chemical cues in *P. ornatus* juveniles warrants consideration for communal culture where populations may be mixed due to size grading or upscaling. Crucially, this distinction allows for a simple bioassay suitable for further research on juvenile response to conspecific moult cues as we investigate the pathways mediating cannibalism of post-moults in culture.

Some cannibalistic crustaceans are risk-averse to conspecific injury and disease cues^6–8,25,26^, while others seek out the injured leading to opportunistic cannibalism^24^. Given this, social relationship and moult-stage may determine if a lobster is averse to a moulting conspecific due to risk or attracted as an opportunity for exuvia consumption or cannibalism. Post-moult and inter-moult lobsters avoiding the moulting cue of conspecifics they are familiar with, may indicate familiar lobsters are knowingly avoiding a situation which attracts inter-moult conspecifics who consume moulted exuvia and are possibly cannibalistic. Similar to other spiny lobster responses to disease and injury cues^7,8,26^, post-moult lobsters, in particular, are potentially avoiding danger while their exoskeleton completes restoration after the moult. Additionally, we have seen inter-moult juveniles showing attraction to naïve moulting cues. This may imply an interest in locating the source, as attraction to cues can often correlate with an increase in foraging activity, consumption of exuvia and possibly cannibalism^24^, which is commonly observed in communally housed spiny lobsters^22,57,58^*.*

The average speed and distance travelled by downstream lobsters increases in response to moulting cues in both lobsters attracted to, or avoidant of the cue. Increased locomotion has been previously seen in crayfish, *Procambarus clarkii,* in response to conspecific odour, regardless of social relationship, sex or dominance status^17^. Both measures of speed and distance, are significantly higher while lobsters are exposed to conspecific moulting cues during the hour following the upstream moult, compared to during the baseline observation hour. Regardless of preference for or against the moulting cue, the behaviour of these lobsters was altered by the presence of this chemical cue, suggesting this response is vital to success, either through avoidance of cannibalism or the acquisition of energy via cannibalism or exuvia consumption. Lobsters held in header tanks upstream display a significantly lower average speed than the experimental controls, indicating juveniles have decreased activity on the night they are moulting, in an environment isolated from conspecifics. Similar results were reported by Lipcius and Herrnkind^34^, who described a steep decline in activity in *P. argus* the day of edcysis, likely due to metabolic needs and water absorption.

This study demonstrates the suitability of a two-current choice flume to assess juvenile *P. ornatus* response to moulting cues and provides a behavioural bioassay for chemosensory investigations. This assay can be used to measure responses to stimuli, such as feed attractants and prey or predator cues. The statistical similarity between the experimental and negative controls demonstrates exposure to chemical cues released by inter-moult conspecifics does not impact their behaviour (current preference, average speed and distance covered). This strengthens our conclusion that the significant differences in these behaviours for treatment groups are driven by their exposure to a moulting conspecific. In combination with physiological and molecular analyses a choice flume bioassay can also examine the pathways mediating response behaviour. The physiological and behavioural processes that occur during ecdysis in crustaceans are modulated by several hormones, initially ecdysteroids, followed by a neuropeptide signalling cascade from the central nervous system^59,60^. The use of live lobsters to introduce a chemical cue precludes concentration–response analysis, however research using a two-current choice flume to examine *P. ornatus* concentration–response to specific chemical cues is possible and advantageous for investigating cues released into the environment during ecdysis.

We hypothesise that olfaction may play a significant role in the identification of moulting conspecifics. If this is correct, it would indicate olfaction, at least partially, mediates behaviours such as exuvia consumption and cannibalism. The aesthetasc-olfactory-lobe pathway is responsible for receiving and inferring chemicals of interest from complex inputs, such as conspecifics, predators, food, and habitat^14^. Ablation of *P. argus* olfactory sensilla has demonstrated that the olfactory pathway is vital in the detection of conspecific blood-borne injury cues^26^ and urine-borne cues^10,12^, which mediate avoidant, aggressive, and aggregation behaviours. Spiny lobsters feature sensory sensilla over much of their body, particularly the antennules, mouthparts, and dactyls, innervated with both chemoreceptor and mechanoreceptor neurons, however, olfactory receptor neurons are only present on the aesthetascs^13,61,62^. Evaluating the role of olfaction in identifying moulting conspecifics can be achieved in future research by functionally interrupting the olfactory receptor neurons of juvenile *P. ornatus* and utilising the attraction/avoidance response in a choice flume as a behavioural bioassay.

## Conclusion

Our novel finding is the correlation between moult cue preference and the intersection of biotic and social factors. We have established a behavioural bioassay using a two-current choice flume to signify avoidance or attraction to conspecific moulting cues, finding post-moult and inter-moult juvenile *P. ornatus* avoid familiar moulting cues, while inter-moult lobsters are attracted to naïve moulting cues. This provides a foundation for future research assessing behavioural response to chemical stimuli, such as conspecific and heterospecific odours, feed attractants, or deterrent signals. Our findings demonstrate the importance of chemical cues in facilitating a behavioural response to a moulting conspecific in juvenile *P. ornatus.* Building from this research will provide insight into the role of chemosensory pathways, such as olfaction, in mediating cannibalism of post-moult juveniles in *P. ornatus* aquaculture.

## Supplementary Information

Supplementary Information 1.

Supplementary Video 1.

## Data Availability

Data presented in this study are available on request from the corresponding author.
