# Supplementary material for: Development of a two-current choice flume behavioural bioassay for juvenile Panulirus ornatus response to moulting cues
Source: Sci Rep. 2022 Dec 12;12:21474. doi: 10.1038/s41598-022-25969-7 (PMC9744895; doi:10.1038/s41598-022-25969-7)
Supplement: Supplementary file 1 — Supplementary Information 1. [file 41598_2022_25969_MOESM1_ESM.docx]

**Development of a two-current choice flume behavioural bioassay for juvenile *Panulirus ornatus* response to moulting cues**

**Tara R. Kelly^1^*, Quinn P. Fitzgibbon^1^, Dean R. Giosio^2^, Andrew J. Trotter^1^, Gregory G. Smith^1^**

^1.^ Institute for Marine and Antarctic Studies (IMAS), University of Tasmania, Private Bag 49, Hobart, TAS 7001, Australia.

^2.^ School of Engineering, University of Tasmania, Hobart, TAS 7000, Australia.

*Corresponding author: t.kelly@utas.edu.au

**Supplementary material**

**Supplementary table 1.** Activity – average speed (cm.sec^-1^) and total distance travelled (cm) – of *Panulirus ornatus* moulting juveniles, and response juveniles in treatment and control replicates during three observation hours. Mean value ± s.e.m.

|  | **Baseline hour** | | | **Hour before moult** | | **Hour after moult** | |
| --- | --- | --- | --- | --- | --- | --- | --- |
|  | **Distance travelled (cm)** | **Average speed (cm.sec^-1^)** | **Distance travelled (cm)** | | **Average speed (cm.sec^-1^)** | **Distance travelled (cm)** | **Average speed (cm.sec^-1^)** |
| **Choice arena lobsters**  **Treatment (n=22)** | 828 ± 112 | 1.8 ± 0.4 | 1240 ± 156 | | 2.4 ± 0.3 | 1314 ± 148 | 2.7 ± 0.4 |
| **Choice arena lobsters**  **Control (n=6)** | 1469 ± 283 | 2.2 ± 0.6 | 1185 ± 247 | | 1.8 ± 0.4 | 1159 ± 430 | 2.01 ± 0.7 |
| **Moulting header tank lobsters**  **Treatment**  **(n=17)** | 432 ± 54 | 0.8 ± 0.1 | 481 ± 23 | | 1.0 ± 0.1 | 482 ± 28 | 1.0 ± 0.1 |


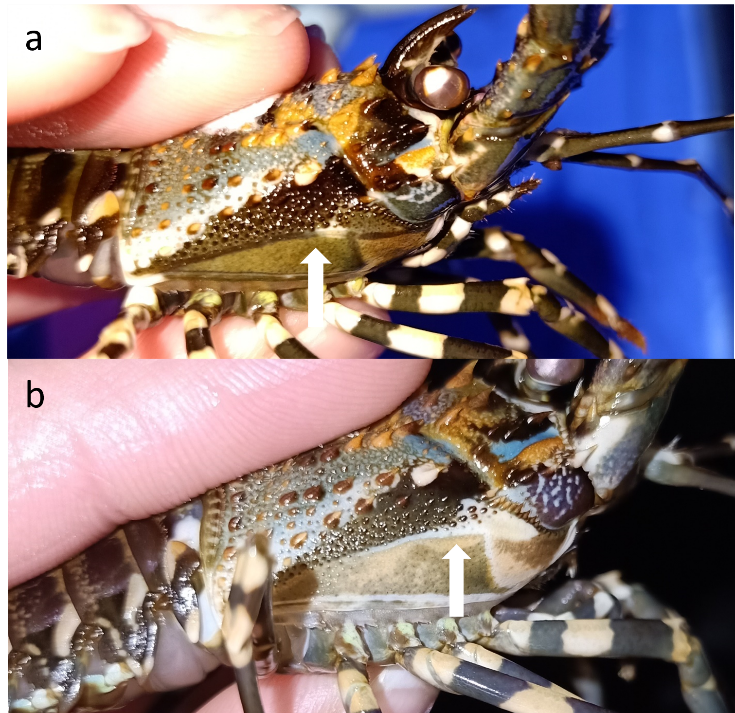


**Supplementary figure 1.** (a) Ecdysial suture line visible on gill cover of J3 instar *P. ornatus*. Photo taken 12-18 h prior to ecdysis. (b) Inter-moult J3 instar *P. ornatus*, no dark suture line visible.


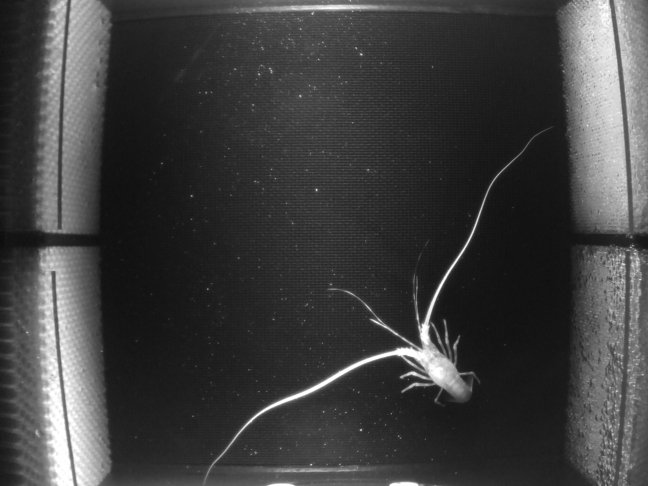

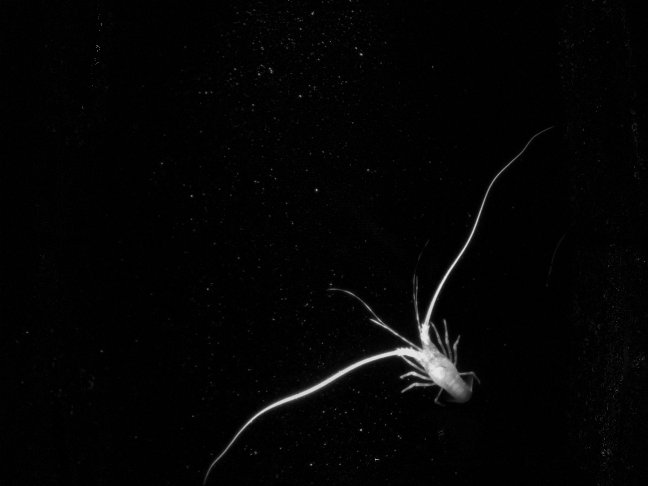


**Supplementary figure 2** Example of background removal using ImageJ AnimalTracker.


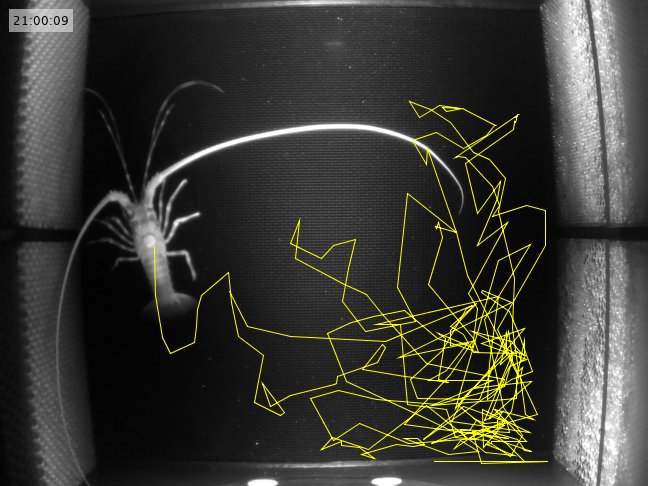


**Supplementary figure 3.** Example track image from ImageJ AnimalTracker following movement of an inter-moult J4 *P. ornatus* in two-current choice flume choice arena.

**Supplementary video 1.** Example track from ImageJ AnimalTracker at 6fps. Following movement of an inter-moult J4 *P. ornatus* in two-current choice flume choice arena.
